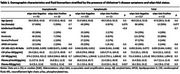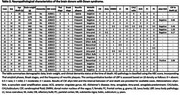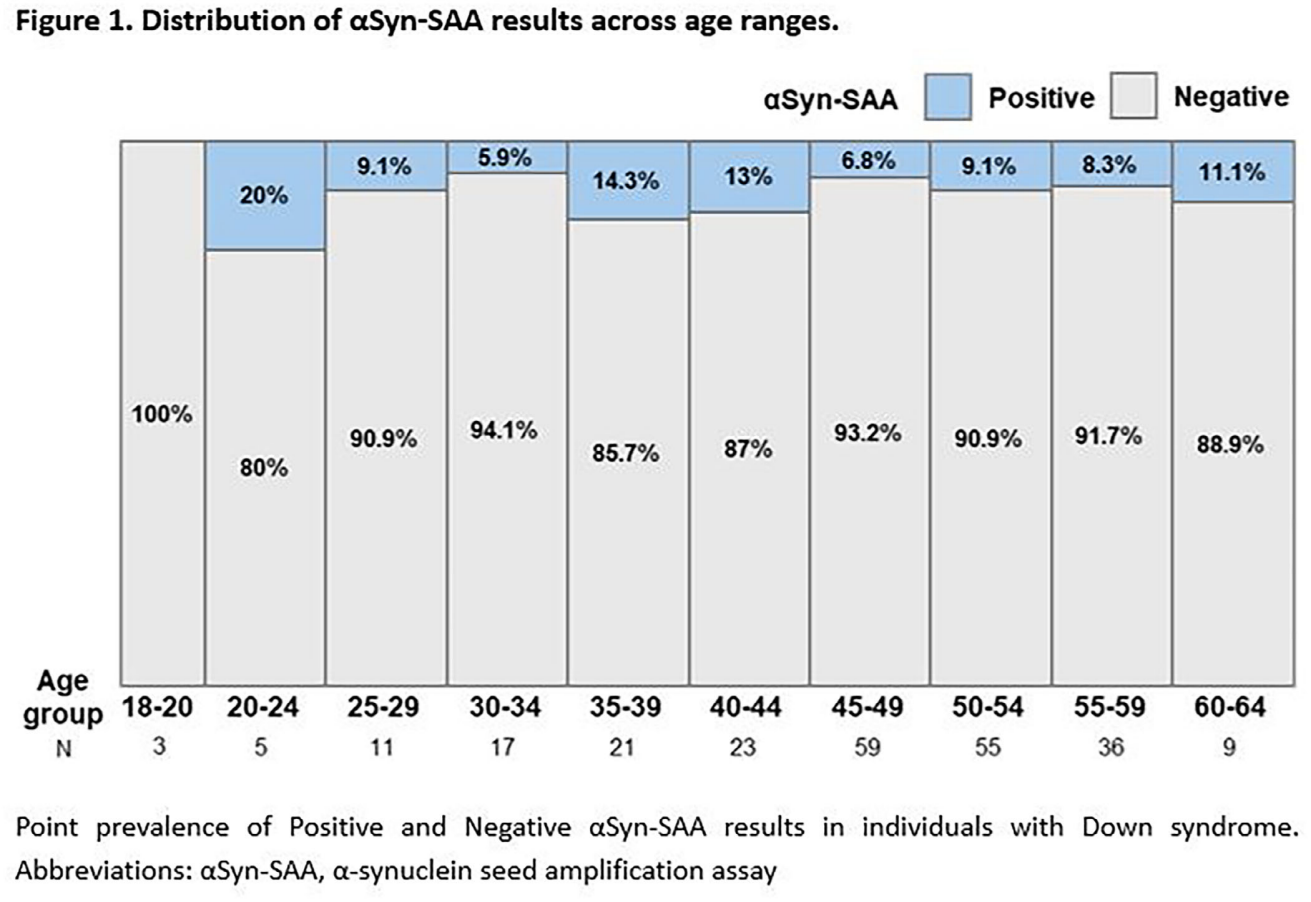# Alpha‐Synuclein Pathology in Down Syndrome‐Associated Alzheimer's Disease: Insights from Seed Amplification Assay and Neuropathology

**DOI:** 10.1002/alz70856_106390

**Published:** 2026-01-07

**Authors:** Íñigo Rodríguez‐Baz, Alexander M Bernhardt, Iban Aldecoa, Javier Arranz, José Enrique Arriola‐Infante, Lucía Maure‐Blesa, Maria Carmona‐Iragui, Sebastian Longen, Svenja Verena Trossbach, Armin Giese, Torsten Matthias, Bessy Benejam, Laura Videla, Laura Del Hoyo, Isabel Barroeta, Aida Sanjuan Hernandez, Susana Fernandez, Lídia Vaqué‐Alcázar, Mateus Rozalem Aranha, Alejandra O. Morcillo‐Nieto, Georg Nübling, Olivia Wagemann, Anna Stockbauer, Mireia Tondo, Alexandre Bejanin, Alberto Lleó, Daniel Alcolea, Laura Molina, Juan Fortea, Johannes Levin

**Affiliations:** ^1^ CIBERNED, Network Center for Biomedical Research in Neurodegenerative Diseases, National Institute of Health Carlos III, Madrid, Spain; ^2^ Sant Pau Memory Unit, Hospital de la Santa Creu i Sant Pau, Institut de Recerca Sant Pau ‐ Universitat Autònoma de Barcelona, Barcelona, Spain; ^3^ German Center for Neurodegenerative Diseases (DZNE), Munich, Bavaria, Germany; ^4^ University Hospital, LMU Munich, Munich, Bavaria, Germany; ^5^ Neurological Tissue Bank of the Biobank ‐ Hospital Clínic ‐ FRCB ‐ IDIBAPS, Barcelona, Spain; ^6^ Pathology Department, Biomedical Diagnostic Center, Hospital Clínic de Barcelona, Barcelona, Spain; ^7^ Barcelona Down Medical Center, Fundació Catalana Síndrome de Down, Barcelona, Spain; ^8^ AESKU. Diagnostics GmbH, Mikroforum Ring 3, 55234, Wendelsheim, Germany; ^9^ MODAG GmbH, Mikroforum Ring 3, 55234, Wendelsheim, Germany; ^10^ Department of Medicine, Faculty of Medicine and Health Sciences, Institute of Neurosciences, University of Barcelona, Barcelona, Spain. Institut d’Investigacions Biomèdiques August Pi i Sunyer (IDIBAPS), Barcelona, Spain; ^11^ Neuroradiology Section, Department of Radiology, Hospital de la Santa Creu i Sant Pau, Biomedical Research Institute Sant Pau, Universitat Autònoma de Barcelona, Spain, Barcelona, Spain; ^12^ Institut d’Investigacions Biomèdiques Sant Pau ‐ Hospital de Sant Pau, Universitat Autònoma de Barcelona, Servei de Bioquímica, Barcelona, Spain; ^13^ Centro de Investigación Biomédica en Red en Diabetes y Enfermedades Metabólicas, CIBERDEM, Barcelona, Barcelona, Spain; ^14^ Alzheimer's disease and other cognitive disorders Unit. Hospital Clínic de Barcelona; FRCB‐IDIBAPS; University of Barcelona, Barcelona, Spain; ^15^ Munich Cluster for Systems Neurology (SyNergy), Munich, Munich, Germany

## Abstract

**Background:**

Down syndrome (DS) is a genetic cause of Alzheimer's disease (AD), with virtually all individuals developing AD pathology by their fourth decade due to *Amyloid Precursor Protein (APP)* gene overexpression. In addition to amyloid beta (Aβ) plaques and hyperphosphorylated tau (*p*‐Tau) aggregates, DS‐associated AD (DSAD) often includes α‐synuclein (αSyn) aggregates, contributing to Lewy body pathology (LBP). The αSyn Seed Amplification Assay (SAA) in cerebrospinal fluid (CSF) enables the *in vivo* detection of misfolded αSyn. While αSyn‐SAA has revealed αSyn pathology in autosomal dominant (6%–11%) and sporadic (21%–45%) AD, its role in DSAD remains unexplored. This study investigates αSyn‐SAA positivity in DSAD, linking *in vivo* findings to fluid biomarkers and neuropathology.

**Method:**

We analyzed CSF of 270 adults with DS from the DABNI and AD21 cohorts by αSyn‐SAA, encompassing asymptomatic and symptomatic AD stages (prodromal/dementia). Additional biomarkers included CSF Aβ1‐42/1‐40, CSF and plasma *p*‐Tau181 and neurofilament light chain (NfL) levels. Neuropathological evaluations in 19 brain donors, including 5 with antemortem CSF, assessed AD neuropathology and LBP.

**Result:**

As shown in Table 1, αSyn‐SAA positivity was observed in 9.2% of participants, consistent across age groups (Figure 1) and cognitive stages. Symptomatic αSyn‐SAA‐positive individuals exhibited significantly higher plasma NfL levels compared to αSyn‐SAA‐negative individuals (31.0 vs. 21.1 pg/mL, *p* =  0.027). Neuropathological analysis revealed LBP in 47% of cases, with the amygdala and olfactory bulb being the most frequently affected regions (Table 2). Among the five donors with antemortem CSF, the only αSyn‐SAA‐positive case corresponded to an individual with severe neocortical LBP.

**Conclusion:**

This study examines the relationship between LBP and DSAD, identifying a prevalence of αSyn‐SAA positivity comparable to autosomal dominant AD but lower than sporadic AD. A potential association was noted between severe neocortical LBP and αSyn seeding activity, while age and cognitive status did not significantly influence positivity rates. Misfolded αSyn was detectable from early ages in individuals with DS. Further research is required to elucidate the mechanisms underlying LBP in DSAD, assess its clinical impact on cognitive and motor symptoms, and explore relationships with other biomarkers.